# Safety, tolerability, pharmacokinetics and pharmacodynamics of a novel farnesoid X receptor (FXR) agonist-TQA3526 in healthy Chinese volunteers: a double-blind, randomized, placebo-controlled, dose-escalation, food effect phase I study

**DOI:** 10.1080/07853890.2023.2264850

**Published:** 2023-12-10

**Authors:** Jia Xu, Hong Zhang, Hong Chen, Xiaoxue Zhu, Haiyan Jia, Zhongnan Xu, Dandan Huo, Hong Zhang, Cuiyun Li, Yanhua Ding

**Affiliations:** a Phase I Clinical Trial Unit, First Hospital, Jilin University, Changchun, China; b Chia Tai Tianqing Pharmaceutical Group Co. Ltd., Nanjing, China

**Keywords:** TQA3526, pharmacokinetics, pharmacodynamics, safety, tolerability, food effect

## Abstract

***Background:*** TQA3526 is a novel farnesoid X receptor agonist developed to treat non-alcoholic steatohepatitis (NASH) or primary biliary cholangitis (PBC). This study aimed to evaluate the safety, tolerability, pharmacokinetics (PK), and pharmacodynamics (PD) of TQA3526 in healthy Chinese patients.

***Methods:*** Healthy subjects aged 18–55 years were enrolled in this double-blinded, first-in-human, placebo-controlled single ascending dose (1, 2, 5, and 10 mg) comprising food effect investigation (10 mg) and multiple dose study (2 mg and 0.2 + 0.5 + 1 mg). Safety was assessed on the basis of adverse events. The TQA3526 concentrations were analysed in the PK study. Alkaline phosphatase (ALP), fibroblast growth factor-19 (FGF19), bile acid precursor C4 (7α-hydroxy-cholest-4-ene-3-one), cholesterol, and bile acid were selected for PD analysis.

***Results:*** TQA3526 was well tolerated, and the primary adverse drug reaction was pruritus, as expected. The exposure to TQA3526 increased in a dose-dependent manner after a single dose of 1–10 mg. The exposure was higher after food intake. A steady state was reached around 5 days, and obvious plasma accumulation of TQA3526 was observed in the multiple dose study. TQA3526 increased circulating FGF-19 and decreased C4 levels in a dose-dependent manner. ALP increased only mildly in the 2 mg multiple dose cohort.

***Conclusions:*** TQA3526 (<10 mg/day) was safe and tolerable in healthy Chinese subjects. The safety profile and PK/PD characteristics of TQA3526 support further evaluation of patients with NASH or PBC. This study was registered at https://www.chictr.org.cn/ under the identifier ChiCTR1800019570.

## Introduction

Non-alcoholic fatty liver disease (NAFLD) is a common hepatic disorder with a prevalence in Western and Asian countries of 20%–30% [1], which can progress to non-alcoholic steatohepatitis (NASH), cirrhosis, and hepatocellular cancer (HCC). NASH is rapidly becoming the leading cause of end-stage liver disease and liver transplantation [2, 3]. The NAFLD prevalence was estimated to increase 29.1% from 246.33 million cases in 2016 to 314.58 million NAFLD cases in 2030 for China [4].

Studies have shown that most patients have no previous symptoms or diagnosis [5]. NAFLD is characterized by the accumulation of fat and inflammation in the liver [6–8], The exact cause of NAFLD progression is unknown, but obesity, high blood pressure, and diabetes can promote its progression [9, 10]. Based on its pathogenesis, its therapy can be divided into three approaches: anti-inflammatory, anti-fibrosis, and anti-metabolic syndrome.

Dietary modification, exercise, and management of comorbidities have guidelines for the management of NASH/NAFLD which were published by the American Association for the Study of Liver Diseases (AASLD) [11], Japan Society of Hepatology (JSH) [12], and the European Association for the Study of Diabetes (EASL) [13]. However, diet and lifestyle intervention measures cannot be implemented successfully or sustainably in most patients [14].

Pharmacological therapies for NASH treatment are widely investigated such as insulin sensitizers (metformin and pioglitazone), antioxidants (vitamin E and pentoxifylline), cholesterol-lowering drugs (statins and aramchol), farnesoid X receptor (FXR) agonists (obeticholic acid [OCA] and tropifexor), peroxisome proliferator-activated receptors (PPARs; elafibranor and saroglitazar), thyroid hormone receptor (THR; resmetirom), and glucagon-like peptide (GLP)-1 agonist (liraglutide and semaglutide).

Pioglitazone and vitamin E are recommended for NASH treatment, although their long-term efficacy and safety should be established [15]. However, long-term or high-dose vitamin E treatment may increase the risk of prostatic cancer [16], and haemorrhagic stroke [17]. In addition, the side effects of pioglitazone on weight gain and risk of congestive heart failure may limit its use in NASH treatment [18].

FXR, a nuclear receptor, regulates metabolic pathways, including glucose homeostasis and inflammatory and fibrogenic processes. Activation of FXR expression is inversely correlated with NASH severity of NASH [19]. OCA has proven to be effective in the treatment of NASH with liver fibrosis [20], while its pruritus and a significant increase in low-density lipoprotein (LDL) cholesterol concerns [21]. Resolution of NASH was not attained in any of the patients.

The original indication for OCA is primary biliary cholangitis (PBC). PBC, also known as primary biliary cirrhosis, is an autoimmune liver disease that can cause biliary epithelial cell injury, cholestasis, fibrosis, and biliary cirrhosis. FXR activation treats PBC by depressing cholesterol 7α-hydroxylase/bile acid transporters, inducing the production of fibroblast growth factor-19 (FGF-19), a negative regulator of bile acid synthesis, and up-regulating organic solute transporters and the bile salt export pump.

TQA3526, developed by Chia Tai Tianqing Pharmaceutical Group Co. Ltd. (Nanjing, China), is a novel FXR developed for use in patients with NASH and/or PBC. TQA3526 is a highly selected TGR5/FXR that is predicted to reduce pruritus compared to OCA (TGR5/FXR ratio of 1:193). The half-maximal effective concentration (EC_50_) of TQA3526 for FXR was only 1/100 of that of OCA *in vitro* (3.1 vs. 385.0 nM). *In vivo* efficacy studies conducted in QA3526 showed better improvement in NAS score, liver fibrosis, and transaminase ta carbon tetrachloride (CCl_4_)-induced mouse model of acute fibrosis showed that Than OCA. In an ANIT mouse model of cholestasis, TQA3526 significantly decreased total bile acid and bilirubin levels. Compared to OCA, TQA3526 has shown better therapeutic efficacy and fewer side effects in preclinical studies. TQA3526 could have the same efficacy as 1/5–1/10 dosage of OCA to overcome the disadvantages of OCA’s exorbitant price and side effects of OCA. TQA3526 has a safety profile that warrants human studies based on safety and toxicological data from rat, mouse, and dog studies.

This Phase I first-in-human study was designed to evaluate the safety, tolerability, pharmacokinetics (PK), and pharmacodynamics (PD) of TQA3526 in healthy Chinese volunteers. Furthermore, this study was used to help select the optimal dosages for subsequent phase 1b and phase 2 studies in patients with NASH or PBC.

## Materials and methods

### Drugs

The formulations were 0.1 mg, 0.5, 1 and 5 mg tablets of TQA3526, manufactured by Frontage Laboratories (Nanjing, China) Co., Ltd. (Lot Number: 19080701; 19080702; 180803301; 180831201; Expiration Date: 6 August 2021; 6 August 2021; 29 August 2020; 30 August 2020). The placebo tablet was also manufactured by Frontage Laboratories (Nanjing) Co., Ltd. (Lot Number: 180830101; expiration date: 29 August 2020). These formulations were provided by Chia Tai Tianqing Pharmaceutical Group Co., Ltd. (Nanjing, China).

### Study population

Healthy Chinese males and females aged 18–55 years with a body mass index (BMI) of 18–28 kg/m^2^ and normal alanine transaminase, aspartate aminotransferase (AST), and alkaline phosphatase (ALP) levels were enrolled in this study. Health status was determined by medical history, physical examination, laboratory tests, abdominal ultrasonography, and electrocardiography. Females with child-bearing potential were instructed to use appropriate contraception throughout the study and for at least 6 months after the last dose of study medication. Participants in any clinical study 3 months prior to screening, with a history of gastrointestinal or biliary system disease, were excluded. Study participants were excluded if they had taken any prescription medication or herbal medicine within 2 weeks.

This study was carried out in accordance with the recommendations of Good Clinical Practice and the Declaration of Helsinki. The study protocol was approved by the Ethics Committee of the First Hospital of Jilin University, Changchun, Jilin, China. All participants provided written informed consent in accordance with the Declaration of Helsinki, and the clinical trial registration number was ChiCTR1800019570 (https://www.chictr.org.cn/).

### Study design

This study was conducted using a randomized (in each dose cohort), double-blind, placebo-controlled, dose-escalation design consisting of a single ascending dose (SAD; Part 1), multiple doses (Part 2), and food effect (Part 3). In part 1, the subjects randomly received a single dose of TQA3526 or placebo in the fasted state after an overnight fast at a ratio of 2:0 (1 mg) or 8:2 (2, 5, and 10 mg). Furthermore, subjects in the 10 mg cohort repeated the study under the fed state after a 10- or 19-day washout period to assess the effect of a high-fat diet on the PK profile of TQA3526 as part 3. Part 2 contained two cohorts. In the ninth cohort, study participants randomly received multiple oral doses of 2 mg TQA3526 or placebo once daily for 7 days under the fasted state at a ratio of 8:4. In the tenth cohort, study participants randomly received continuous multiple oral doses of 0.2 mg (10 days), 0.5 mg (10 days) and 1 mg (10 days) TQA3526 or placebo once daily at a ratio of 8:4.

Subjects were admitted to the Clinical Trial Center the day before drug administration and discharged 72 h after the last drug administration. During the confinement period, the subjects were provided standardized meals (except for the high-fat diet in the 10 mg dose cohort) to minimize the effect of food on PK/PD evaluations.

For the SAD study, a maximum recommended starting dose of 105 mg was calculated based on the standard 10-fold safety margin from the beagle dog no-observed-adverse-effect-level (NOAEL) exposure study, per the regulatory guidelines. However, 0.5, 1, and 2 mg were estimated to be effective doses, as TQA3526 could have the same efficacy as 1/5–1/10 dosage of OCA (5 or 10 mg/day) based on preclinical efficacy studies. Therefore, a single dose range of 1–50 mg was chosen, and multiple doses were set at 2 mg and 5 + 10 + 20 mg originally. As most subjects experienced pruritus after multiple doses of 2 mg, the single dose range was adjusted to 1–10 mg and 5 + 10 + 20 mg was replaced by 0.2 + 0.5 + 1 mg for safety concerns.

### Pharmacokinetic and pharmacodynamic assessments

Blood samples (4 mL) for PK analysis were obtained in chilled collection tubes containing the anticoagulant K_2_-ethylenediaminetetraacetic acid (EDTA) at 0 h (pre-dose), and 10 min, 20 min, 30 min, 45 min, 1 h, 1.5 h, 2 h, 3 h, 4 h, 6 h, 8 h, 12 h (day 1), 24 h (D2), 48 h (D3), 72 h (D4) in parts 1 and 3. For part 2, the ninth cohort, blood samples (4 mL) were obtained at 0 h (pre-dose), 10 min, 20 min, 30 min, 45 min, 1 h, 1.5 h, 2 h, 3 h, 4 h, 6 h, 8 h, 12 h (D1 and D7), D2–D6, and D8–D10. The tenth cohort, blood samples (4 mL) were obtained at 0 h (pre-dose), and 10 min, 20 min, 30 min, 45 min, 1 h, 1.5 h, 2 h, 3 h, 4 h, 6 h, 8 h, 12 h (D20 and D30), D8–D10, D15, D17, D19, D21, D25, D27, D29, and D31–D33. Samples were centrifuged for 15 min (2–8 °C 1800 g) for plasma separation. For the food effect study, urine samples were collected 0 h, 0–6 h, 6–12 h, 12–24 h, 24–48 h, and 48–72 h after dosing. Faecal samples were collected at 0–72 h post-dose. All of the samples were maintained at −80 °C until analysis.

The plasma samples were analysed for TQA3526 using a validated analytical method based on liquid chromatography-tandem mass spectrometry (LC-MS/MS) using human plasma, urine, and faecal samples. The linearity ranges were 0.2–200 ng/mL for TQA3526 in the plasma. The precision rate was ≤5.7%, with an accuracy in the range of −0.6 to 1.7%.

ALP, FGF19, bile acid precursor C4 (7α-hydroxy-cholest-4-ene-3-one), cholesterol, and bile acid were selected for PD analysis. PD blood samples were obtained at 0 h (pre-dose), and 2 h, 4 h, 6 h, 8 h, 12 h (D1), 24 h (D2), 72 h (D4) in parts 1 and 3. For part 2, the ninth cohort, blood samples were obtained at 0 h (pre-dose), and 2 h, 4 h, 6 h, 8 h, 12 h (D1 and D7), D2, D3, D5, D8, and D9. In the tenth cohort, FGF19 was obtained at 0 h (pre-dose), and 10 min, 20 min, 30 min, 45 min, 1 h, 1.5 h, 2 h, 3 h, 4 h, 6 h, 8 h, 12 h (D20 and D30), D8–D10, D15, D17, D19, D21, D25, D27, D29, and D31–D33.

Serum ALP levels were measured using a Beckman Coulter kit assay with neryl diphosphate (NPP) as substrates and 2-amino-2-methyl-1-propanol (AMP) as buffers. Cholesterol activity was measured using a Beckman Coulter test kit with enzyme method. Total bile acid activity was measured using a Beckman Coulter kit with circulating enzymatic method. FGF-19 was tested using a quantification assay by an ELISA kit. C4 was analysed using a validated analytical method based on LC-MS/MS.

### Pharmacokinetic and pharmacodynamic analysis

The sample size was determined based on the Chinese regulatory requirement for a phase I study. A total of 64 volunteers were enrolled in this study. The safety population included volunteers who had received at least one dose of the study treatment; the PK and PD population included volunteers who had received at least one dose of the study treatment and for whom PK and PD samples were obtained and analysed.

The PK parameters were calculated with WinNonlin 8.3.1 (Certara, Princeton, NJ, USA) using non-compartmental analysis, including *T*_max_, *C*_max_, *t*_1/2_, AUC_0–24_, AUC_0–72_, AUC_0–_*_t_*, AUC_0–∞_, AUC__%Extrap_, CL/F, V*_z_*/F, *T*_max,ss_, *C*_min,ss_, *C*_max,ss_, *t*_1/2,ss_, AUC_0–∞,ss_, AUC_0–72,ss_, AUC_tau,ss_, CL/F_,ss_, V*_z_*/F_,ss_, *R*_ac_ and DF. The amount (Ae), apparent fraction (Fe), and renal clearance (CLR) of TQA3526 were calculated using the SAS software (version 9.4, SAS Institute, Cary, NC, USA).

To examine dose proportionality, a power model, log (parameter) = α + β × log(dose), where α and β are constants, was fitted to the data. The slope (β), with its corresponding 90% confidence interval (CIs), was estimated to assess the degree of dose proportionality. A β value of 1 indicates linear PKs.

In the food effect study, *C*_max_ and AUC_0–∞_ were compared between the fasted and fed states using a mixed-effects model for log-transformed PK values with a treatment sequence, period, and treatment as fixed effects, and subjects nested with a sequence fitted as a random effect. The geometric mean ratios of the two drugs were calculated by exponentiation of the differences in the least-squares mean (LSM) along with the corresponding 90% CIs. The absence of a food effect was true if 90% of the CIs were within the 80%–125% equivalence interval.

The PD parameters of *C*_min_ or *C*_max_ for baseline-adjusted ALP [22], FGF19, BA precursor C4 (7α-hydroxy-cholest-4-ene-3-one) [23], cholesterol, and bile acid were observed in the curves. Changes in FGF-19 relative to baseline depended on TQA3526 concentration and could be fitted to an *E*_max_ model to predict the maximum efficacy (*E*_max_) and EC_50_ [24].

### Safety and tolerability assessments

Safety evaluation included monitoring of adverse events (AEs) and serious AEs (SAEs), clinical laboratory tests (hematology, clinical chemistry, and routine urinalysis), vital signs, electrocardiograms (ECGs), and physical examinations until the 72 h follow-up contact. All relevant information regarding the AE/SAE was recorded using the appropriate data collection tool. The severity of AEs was assessed according to the Common Terminology Criteria for Adverse Events (CTCAE 5.0).

## Results

### Volunteers

A total of 64 out of 373 screened Chinese subjects were enrolled in this study and completed the safety analysis after participation in the study. 58 volunteers completed the study. Six participants withdrew from the study for personal reasons. The demographic characteristics and baselines for clinical chemistry tests of the volunteers are summarized in Table 1.

**Table 1.** Demographics of the subject in this study.

Cohort	Single ascending dose				Food effect	Multiple ascending doses		Placebo (*N* = 14)
	1 mg (*N* = 2)	2 mg (*N* = 8)	5 mg (*N* = 8)	10 mg (*N* = 8)	10 mg (*N* = 16)	2 mg (*N* = 8)	0.2 mg + 0.5 mg + 1 mg (*N* = 8)	
Age (years)	36.0 ± 14.14	38.6 ± 8.25	35.4 ± 8.94	33.0 ± 4.66	34.7 ± 6.58	36.5 ± 7.84	35.6 ± 7.05	37.1 ± 8.28
Gender								
Male	1 (50.00)	4 (50.00)	4 (50.00)	4 (50.00)	8 (50.00)	4 (50.00)	4 (50.00)	7 (50.00)
Female	1 (50.00)	4 (50.00)	4 (50.00)	4 (50.00)	8 (50.00)	4 (50.00)	4 (50.00)	7 (50.00)
Height (cm)	159.05 ± 6.576	165.55 ± 7.260	163.70 ± 8.407	162.39 ± 7.685	162.11 ± 7.888	163.44 ± 9.562	163.60 ± 10.012	162.70 ± 7.925
Weight (kg)	55.40 ± 6.505	63.49 ± 5.518	62.31 ± 9.873	61.65 ± 9.879	63.14 ± 10.050	61.20 ± 4.711	64.31 ± 8.207	65.08 ± 8.979
BMI (kg/m^2^)	21.5 ± 0.71	23.3 ± 2.55	23.3 ± 2.71	23.3 ± 3.20	23.9 ± 2.67	23.0 ± 1.60	24.1 ± 3.18	24.6 ± 1.65
Alanine aminotransferase (U/L)	12.25 ± 5.445	18.56 ± 9.913	17.69 ± 9.577	17.66 ± 8.710	18.95 ± 8.648	14.43 ± 5.213	15.19 ± 8.693	18.69 ± 7.440
Aspartate aminotransferase (U/L)	25.65 ± 2.899	21.25 ± 4.080	18.84 ± 4.760	19.58 ± 4.585	20.96 ± 4.332	16.65 ± 4.313	20.13 ± 5.897	20.08 ± 3.950
Alkaline phosphatase (U/L)	50.10 ± 3.111	78.48 ± 18.981	70.91 ± 21.073	73.03 ± 11.949	68.54 ± 17.465	61.79 ± 24.903	54.33 ± 10.581	60.90 ± 21.287
Cholesterol (mmol/L)	4.120 ± 0.1838	4.936 ± 0.9839	4.538 ± 0.5628	4.353 ± 0.5093	4.388 ± 0.4564	4.144 ± 0.7260	3.844 ± 0.3836	4.725 ± 0.8766
Bile acid (umol/L)	7.40 ± 2.121	3.20 ± 1.869	3.35 ± 2.766	4.60 ± 2.210	3.95 ± 3.119	3.21 ± 2.677	3.79 ± 3.030	3.58 ± 1.837
Glucose (mmol/L)	5.300 ± 0.1697	4.621 ± 0.2469	4.840 ± 0.3153	4.706 ± 0.4109	4.652 ± 0.4051	4.609 ± 0.4303	5.173 ± 0.2691	4.784 ± 0.3458

Data are mean ± *SD* or n (%). BMI: body mass index.

### Tolerability and safety

The formal maximum tolerated dose (MTD) was 10 mg and dose-limiting toxicity (DLT) was pruritus for TQA3526. No severe or serious AEs were observed in any subject. The safety results are presented in Table 2.

**Table 2.** Adverse events incidence after administration of TQA3526 by dose cohort (*n* = 74).

Cohort	Single ascending dose					Food effect				Multiple doses		
	1 mg (*N* = 2)	2 mg (*N* = 8)	5 mg (*N* = 8)	10 mg (*N* = 8)	Placebo (*N* = 6)	10 mg Fasted (*N* = 16)	10 mg Fed (*N* = 16)	Placebo Fasted (*N* = 2)	Placebo Fed (*N* = 2)	2 mg (*N* = 8)	0.2 mg + 0.5 mg + 1 mg (*N* = 8)	Placebo (*N* = 8)
AE	0	4 (50.00)	5 (62.50)	7 (87.50)	1 (16.67)	13 (81.25)	16 (100)	1 (50.00)	2 (100)	8 (100)	8 (100)	5 (62.50)
ADR	0	4 (50.00)	5 (62.50)	7 (87.50)	1 (16.67)	13 (81.25)	15 (93.75)	1 (50.00)	2 (100)	8 (100)	8 (100)	4 (50.00)
AE (grade ≥3)^a^	0	1 (12.50)	1 (12.50)	0	0	2 (12.50)	9 (56.25)	0	1 (50.00)	7 (87.50)	5 (62.50)	0
ADR (grade ≥3)	0	1 (12.50)	1 (12.50)	0	0	2 (12.50)	9 (56.25)	0	1 (50.00)	7 (87.50)	5 (62.50)	0
SAE	0	0	0	0	0	0	0	0	0	0	0	0
Death	0	0	0	0	0	0	0	0	0	0	0	0
ADR occurring in at least two subjects												
Hypocalcaemia	0	2 (25.00)	0	0	0	0	0	0	0	0	0	0
White blood cell decrease	0	1 (12.50)	2 (25.00)	1 (12.50)	0	2 (12.50)	1 (6.25)	0	0	1 (12.50)	2 (25.00)	0
Neutrophil count decrease	0	1 (12.50)	3 (37.50)	2 (25.00)	0	3 (18.75)	3 (18.75)	0	1 (50.00)	1 (12.50)	2 (25.00)	0
Alanine aminotransferase increase	0	0	0	1 (12.50)	0	2 (12.50)	2 (12.50)	0	0	4 (50.00)	3 (37.50)	1 (12.50)
Aspartate aminotransferase increase	0	0	0	1 (12.50)	0	2 (12.50)	4 (25.00)	0	0	4 (50.00)	5 (62.50)	0
Thyroid-stimulating hormone decrease	0	0	1 (12.50)	1 (12.50)	0	1 (6.25)	0	0	0	0	0	0
Bile acid increase	0	0	0	0	0	0	1 (6.25)	0	0	1 (12.50)	0	0
Arthralgia	0	0	0	1 (12.50)	0	1 (6.25)	0	0	0	0	0	0
Pruritus	0	1 (12.50)	2 (25.00)	7 (87.50)	1 (16.67)	13 (81.25)	15 (93.75)	1 (50.00)	1 (50.00)	8 (100)	8 (100)	1 (12.50)
Vomiting	0	0	0	0	0	0	0	0	0	2 (25.00)	0	0
Diarrhea	0	0	0	0	0	0	1 (6.25)	0	0	1 (12.50)	0	1 (12.50)
Supraventricular arrhythmia	0	0	0	1 (12.50)	0	1 (6.25)	1 (6.25)	0	0	1 (12.50)	0	0

^a^The Common Terminology Criteria for Adverse Events (CTCAE 5.0) was used to grade adverse events (grades 1–5). Data are *n* (%). AE: adverse event; ADR: adverse drug reaction; *N*: number of subjects analysed.

In the SAD study, 34 AEs were reported by 17 subjects (53.12%), including 16 in the TQA3526 cohort (61.54%) and one in the placebo cohort (16.67%); all AEs were adverse drug reactions (ADRs). In total, two subjects (7.69%) experienced AEs classified as grade 3 in the 2 mg and 5 mg cohorts, respectively. Grade 3 ADRs of neutrophil count decrease were present in two subjects who received 2 and 5 mg of TQA3526. The most frequent AEs were pruritus (42.30%), decreased neutrophil count (23.08%), and decreased white blood cell count (15.38%). Subjects in the higher dose cohorts showed more severe pruritus with higher incidence rates, suggesting that pruritus was dose-dependent. The decrease in leukocyte and neutrophil counts showed self-healing and no dose-related trend. In the food effect cohort, 60 AEs were reported by 15 participants. Fed conditions showed less tolerance than fasted conditions, as nine and two subjects experienced grade 3 pruritus under fed and fasted conditions, respectively. A grade 3 neutrophil count decrease was also observed in one subject who received placebo.

In the multiple dose study, 70 AEs were reported by 21 subjects (87.50%), including 16 in the TQA3526 cohort (100%) and five in the placebo cohort (62.50%), of which 65 AEs were ADRs. Seven (87.50%) and five subjects (62.50%) experienced grade 3 AEs in the 2 mg and 0.2 + 0.5 + 1 mg cohorts, respectively. Primary grade 3 ADRs, pruritus, were present in seven (87.50%) and four (50.00%) subjects who received 2 mg and 0.2 + 0.5 + 1 mg TQA3526. The pruritis was not associated with rash. The most frequent AEs were pruritus (100%), AST increase (56.25%), alanine aminotransferase increase (50%), neutrophil count decrease (18.75%), and white blood cell count decrease (18.75%).

### PK of TQA3526

#### Single ascending dose study and food effect study

**Figure 1.**
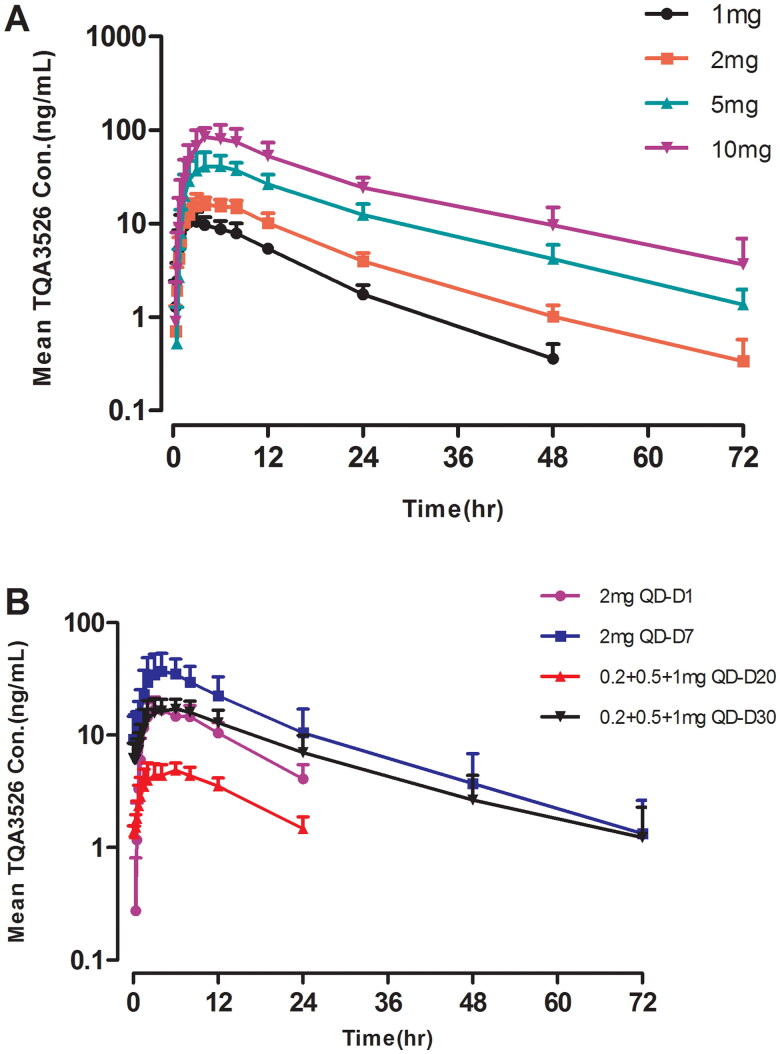
Mean (±*SD*) TQA3526 concentrations (ng/mL) versus time profiles (A) in the single ascending dose study and (B) the multiple dose study.

Data collected from the participants were included in the PK and statistical analyses. The actual time of sample collection was used for statistical calculations. The mean TQA3526 plasma concentration-time profiles after a single dose are shown in Figure 1(A). The PK parameters of TQA3526 obtained from non-compartmental analysis are summarized in Table 3. Following the administration of a single dose, TQA3526 was rapidly absorbed, with its plasma concentrations reaching *C*_max_ between 2.50 and 4.00 h. The mean terminal phase elimination half life (*t*_1/2_) of TQA3526 ranged from 8.92 to 16.69 h with increased values for higher doses. The *C*_max_ of TQA3526 increased from 10.92 to 94.62 ng/mL in the dose range of 1 mg to 10 mg. The mean CL value was similar across all doses. Estimates of the power model exponent β were approximately one, which would be expected under dose proportionality, with estimated values and 95% CIs of 0.9982 (0.8957, 1.1007) for *C*_max_ and 1.0722 (0.9571, 1.1873) for AUC_0–∞_. Hence, PK parameters were linear for TQA3526.

**Table 3.** Plasma pharmacokinetic parameters in the single ascending dose study.

Cohort	*t*_1/2_	*T*_max_	*C*_max_	AUC_0–72 h_	AUC_0–∞_	V*_z_*/F	CL/F
	(h)	(h)	(ng/mL)	(h·ng/mL)	(h·ng/mL)	(L)	(L/h)
Single ascending dose study							
1 mg (*n* = 8)	8.92 ± 0.57	2.50 (2.00–3.00)	10.92 ± 3.50	158.08 ± 40.90	158.93 ± 41.44	83.12 ± 16.46	6.51 ± 1.70
2 mg (*n* = 8)	12.90 ± 2.66	4.00 (2.00–8.00)	17.38 ± 2.83	297.92 ± 60.74	305.27 ± 63.67	124.02 ± 24.06	6.81 ± 1.44
5 mg (*n* = 8)	14.54 ± 2.40	4.00 (3.00–6.00)	48.74 ± 11.13	841.48 ± 227.83	871.24 ± 240.94	125.35 ± 27.57	6.04 ± 1.30
10 mg (*n* = 8)	16.69 ± 6.36	4.00 (3.00–6.00)	94.62 ± 30.01	1704.04 ± 566.46	1817.85 ± 681.73	134.45 ± 24.38	6.07 ± 1.30
Food effect study (10 mg)							
Fasted (*n* = 14)	16.23 ± 5.55	4.00 (3.00–8.00)	97.70 ± 27.50	1889.28 ± 700.71	2014.64 ± 821.70	122.90 ± 29.06	5.74 ± 2.14
Fed (*n* = 16)	16.78 ± 3.56	5.00 (2.00–8.00)	118.34 ± 29.24	2384.38 ± 766.55	2543.96 ± 887.63	100.54 ± 20.36	4.40 ± 1.52

Data are expressed as mean ± *SD* unless otherwise specified. *T*_max_ is expressed as median (range).

In the food effect study, the mean total exposures were higher under the fed condition with a geometric LSM ratio (90% CI) of 122.10% (113.41%–131.45%), indicating that food intake could increase bioavailability. In addition, *T*_max_ was delayed by approximately 1.0 h from 4.0 to 5.0 h under fed conditions, likely due to delays in gastric emptying. The meant_1/2_ and CL/F values were similar under fed and fast conditions.

The *C*_max_ and AUC_0–∞_ values for TQA3526 in females were larger than those in males by approximately 1.22-fold and 1.45-fold under fast and fed conditions, respectively. TQA3526 excretion was low (0.01%) in the urine samples and 33.08% in the faecal samples. TQA3526 was mainly excreted in the faeces.

### Multiple dose study

The mean TQA3526 plasma concentration-time profiles after multiple doses are shown in Figure 1(B). The plasma PK parameters for TQA3526 on days 1 and 7 for the 2 mg cohort and days 20 and 30 for the 0.2 + 0.5 + 1 mg cohort are shown in Table 4. In the 2 mg cohort, the mean *t*_1/2,ss_ and CL/F_ss_ for TQA3526 were 14.78 h and 4.5825 L/h, respectively, while *R*_acc_ values were 2.64, indicating obvious plasma accumulation of TQA3526 on day 7. Steady-state conditions were reached on day 5. The mean steady-state trough concentration of TQA3526 was 9.88 ng/mL. In the 0.2 + 0.5 + 1 mg cohort, the mean *t*_1/2,ss_ and CL/F_ss_ for TQA3526 were 16.80 h and 3.8568 L/h. The mean steady-state trough concentration of TQA3526 was 5.74 ng/mL.

**Table 4.** Plasma pharmacokinetic parameters in the multiple dose study.

Treatment	Day 1			Day 7											
	*T*_max_ (h)	*C*_max_ (ng/mL)	AUC_0–24 h_ (h·ng/mL)	*t*_1/2,ss_ (h)	*T*_max,ss_ (h)	*C*_max,ss_ (ng/mL)	*C*_min,ss_ (ng/mL)	AUC_tau,ss_ (h × ng/mL)	AUC_0–∞,ss_ (h × ng/mL)	AUC_0–72,ss_ (h × ng/mL)	CL/F_ss_ (L/h)	V*_z_*/F_ss_ (L)	*R*_AUC_	DF (%)	
2 mg QD (*n* = 8)	3.00 (3.00 − 6.00)	17.61 ± 4.30	236.26 ± 53.75	14.78 ± 3.59	4.00 (3.00–6.00)	39.74 ± 18.06	9.88 ± 6.08	553.92 ± 271.22	829.20 ± 468.64	790.70 ± 434.32	4.58 ± 2.53	88.45 ± 30.24	2.64 ± 1.22	136.48 ± 21.00	
Treatment	Day 20				Day 30										
	*T*_max,ss_ (h)	*C*_max,ss_ (ng/mL)	*C*_min,ss_ (ng/mL)	AUC_tau,ss_ (h·× ng/mL)	*t*_1/2,ss_ (h)	*T*_max,ss_ (h)	*C*_max,ss_ (ng/mL)	*C*_min,ss_ (ng/mL)	AUC_tau,ss_ (h × ng/mL)	AUC_0–∞,ss_ (h × ng/mL)	AUC_0–72,ss_ (h × ng/mL)	CL/F_ss_ (L/h)	V*_z_*/F_ss_ (L)	–	DF (%)
0.2 + 0.5 + 1 mg QD (*n* = 8)	3.50 (1.50–8.00)	5.32 ± 0.90	1.24 ± 0.18	76.08 ± 14.80	16.80 ± 4.69	4.00 (2.00–6.00)	17.85 ± 4.39	5.74 ± 2.22	288.45 ± 88.14	476.52 ± 207.77	440.48 ± 172.88	3.86 ± 1.61	86.06 ± 13.07	–	105.10 ± 18.80

### PD of TQA3526

**Figure 2.**
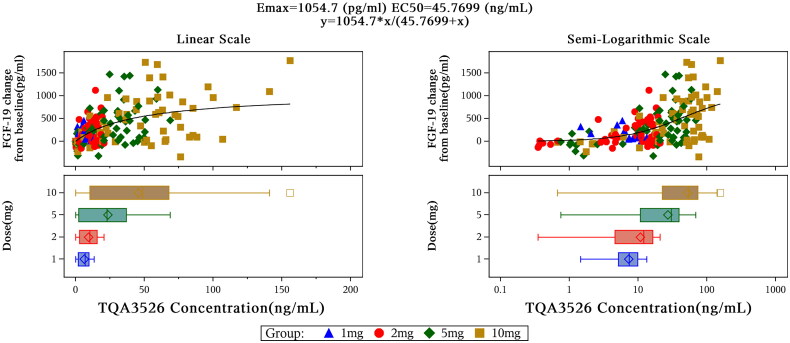
Pharmacokinetic/pharmacodynamic profiles of FGF-19 for the single ascending dose study.

The mean changes in ALP and cholesterol levels were not significant after a single dose of TQA3526 (1–10 mg). The bile acid decrease (*C*_min_ relative to baseline) in the 10 mg cohort was significant. FGF-19 increased (*C*_max_ relative to the baseline) in a dose-dependent manner. Changes of FGF-19 from baseline depending on TQA3526 concentrations could be fitted to an *E*_max_ model (Figure 2), which predicted a maximum efficacy (*E*_max_) at 1054.7 pg/mL and EC_50_ at 45.7699 ng/mL. Although mean C4 decreases were observed, there were no significant differences among the different cohorts, including the placebo cohort. Changes in C4 from the baseline showed a delay in TQA3526 concentration changes in the range of 1–10 mg (Figure 2).

**Figure 3.**
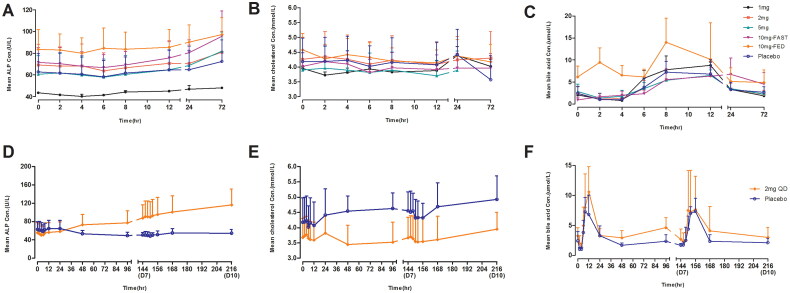
Mean (±*SD*) ALP (U/L), cholesterol (mmol/L), and bile acid (umol/L) concentrations versus time profiles in the single ascending dose study (A–C) and the multiple dose study (D–F).

The mean ALP, bile acid, and cholesterol level changes were not significant after a single dose of 10 mg TQA3526 in the fasted and fed states (Figure 3(A–C)). There were significant FGF-19 increases (*C*_max_ relative to baseline) between TQA3526 treatment cohorts and placebo cohorts, with larger increases in the fed cohort. There was a significant decrease in C4 levels (*C*_min_ relative to baseline) between the treatment and placebo cohorts.

**Figure 4.**
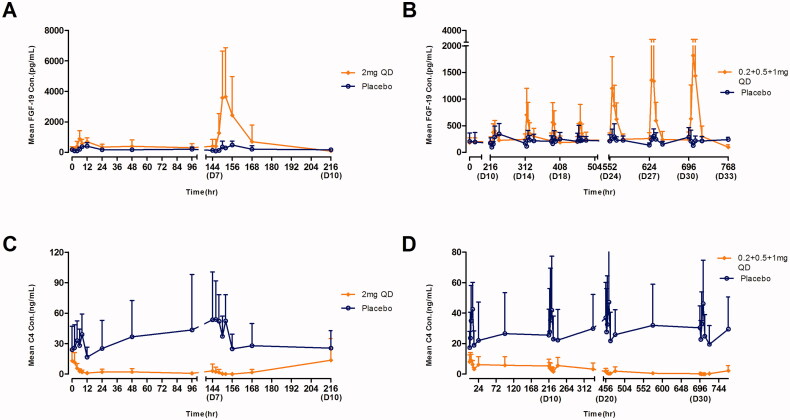
Mean (±*SD*) FGF-19 (pg/mL) and C4 (ng/mL) concentrations versus time profiles in the multiple dose study (A) FGF-19 for 2 mg cohort, (B) FGF-19 for 0.2 + 0.5 + 1 mg, (C) C4 for 2 mg cohort, and (D) C4 for 0.2 + 0.5 + 1 mg.

The mean ALP level gradually increased after multiple doses of 2 mg QD until the end of the study (Figure 3(D)). The mean bile acid and cholesterol concentration-time profiles were similar for the TQA3526 treatment and placebo cohorts (Figure 3(E,F)). The mean FGF-19 and C4 concentration-time profiles after multiple doses are shown in Figure 4. There was a significant increase in FGF-19 levels between the TQA3526 treatment cohort and placebo cohort for the 2 mg cohort, with *C*_max_ reaching on day 7. In the 0.2 + 0.5 + 1 mg cohort, the mean FGF-19 levels reached maximum increases during the 1 mg treatment period. The mean C4 concentration-time profiles for the 2 mg cohort were opposite to those for the placebo cohort. The mean C4 levels gradually decreased after multiple doses of 2 mg QD. Similar to the 2 mg cohort, the mean C4 levels gradually decreased in the 0.2 + 0.5 + 1 mg cohort, with a smaller extent of decrease.

## Discussion

This first-in-human, double-blind, placebo-controlled study evaluated the safety, tolerability, and PK/PD characteristics of TQA3526 in healthy Chinese subjects. The safety results indicated that TQA3526 at doses less than 10 mg/day was tolerated by the healthy subjects. The PK results confirmed that TQA3526 was rapidly absorbed with a dose-proportional increase in the exposure. TQA3526 also increased FGF-19 and decreased C4 levels, indicating FXR activation.

In this study, no serious AEs or deaths occurred. The most frequent ADRs were pruritus, increased AST and alanine aminotransferase levels, decreased neutrophil count, and decreased white blood cell count. Grade 3 pruritus rates reached 56.25%, 87.50%, and 50.00% in the 10 mg fed cohort and 2 mg and 0.2 + 0.5 + 1 mg in the multiple dose study, respectively. However, pruritus was also observed in the placobo cohort, indicating that pruritus may have a psychological component. Pruritus usually occurs on day 2 and is mainly manifested in the limbs, especially the palms and feet, facial region, perianal, and genital organs.

In a PBC patient clinical trial, the incidence rates of pruritus were 38%, 56%, and 68% in the placebo, 5 + 10 mg, and 10 mg cohorts, respectively. The severity of pruritus was lower in the titration cohort than in the 10 mg OCA cohort [25]. As mentioned above, we also chose the titration method in our study. The severity of pruritus was lower in the 0.2 + 0.5 + 1 mg group. Pruritus is expected to be an ADR, potentially due to the activation of cell surface bile acid receptors (GPBAR1) [26]. Bile acid sequestrants (cholestyramine), antihistamines (clarityne), and opioid antagonists (naloxone) were used according to their mechanisms of action.

In our study, grade 3 liver injury occurred in two subjects who received multiple doses of 2 mg and 0.2 + 0.5 + 1 mg. A transient decrease in white cells and neutrophils was observed. It has been reported that OCA can reduce triglyceride levels in liver tissues [27]. In addition, OCA decreases high-density lipoprotein cholesterol and increases LDL cholesterol, independent of the dose [28]. However, TQA3526 did not show these effects.

The PK results of the SAD study confirmed that TQA3526 was rapidly absorbed with a dose-proportional increase in the exposure. In the present study, the maximum dose was 10 mg. The *t*_1/2_ of TQA3526 is 8.92–16.69 h and tends to be suitable for once-daily dosing (QD). In terms of excretion, TQA3526 was mainly excreted in faeces (33.0855%), with barely any renal excretion, suggesting that it undergoes substantial metabolism before its elimination.

A food effect study demonstrated that the absorption of TQA3526 was delayed. *C*_max_, AUC_0–_*_t_* and AUC_0–∞_ increased by 16.52%, 21.41%, and 22.10%, respectively, after a high-fat diet. Food intake prolonged the median *T*_max_ from 4 to 5 h without any impact on its elimination, as indicated by comparable *t*_1/2_ values. Increased exposure resulted in more severe pruritus, so subjects would have better administered TQA3526 under fasted conditions in future studies for safety considerations. Body weight is a factor that might lead to exposure differences in subjects of different genders, as the weights of females were lower than those of males.

*T*_max_ and *t*_1/2_ at steady state after multiple doses were comparable to those after a single dose. Steady state was considered to have been reached by day 5 from the observed concentrations and significant exposure accumulation of TQA3526 with *R*_acc_ of 2.64 over the 7 days of dosing in the 2 mg cohort. In the 0.2 + 0.5 + 1 mg cohort, exposures were less than those in the 2 mg cohort after steady state.

The efficacy of TQA3526 was preliminarily investigated in a small size of subjects in this phase I study. TQA3526 increased circulating FGF-19 levels and decreased C4 levels after a single dose (dose-proportional manner) and multiple doses, demonstrating FXR activation. ALP increased only mildly in the 2 mg multiple dose cohort. ALP and cholestasis levels were not altered following TQA3526 treatment. Gender did not alter the effects of TQA3526 treatment on these markers. Limited by the general phase Ia study design, as only a small sample size of subjects and healthy subjects were enrolled, the safety and efficacy of TQA3526 in NASH and PBC require further clinical trials. As the minimum effective dose was found to be 0.2 mg, the starting dose in the TQA3526 phase II study was set at 0.2 mg considering safety as well.

## Conclusions

TQA3526 (<10 mg/day) was safe and tolerable in healthy Chinese subjects. The exposure to TQA3526 increased in a dose-dependent manner after a single dose of 1–10 mg. The safety profile and PK/PD characteristics of TQA3526 support further evaluation of its safety and efficacy in NASH or PBC patients. The efficacy of TQA3526 (0.2 mg) is currently under investigation in a phase II study.

## Data Availability

Data are not available due to ethical restrictions.
